# Barriers, Facilitators, and Intention to Use AI for Breast Cancer Diagnosis: Mixed Methods Study Among Austrian Physicians With and Without AI Experience

**DOI:** 10.2196/80274

**Published:** 2026-06-09

**Authors:** Marlene Kritz, Erol Holawatsch, Doris Anita Behrens, Walter Hyll

**Affiliations:** 1 Department for Economy and Health University for Continuing Education Krems Krems, Lower Austria Austria; 2 Curtin School of Allied Health, Faculty of Health Sciences Curtin University Perth Australia; 3 School of Mathematics Cardiff University Cardiff, Wales United Kingdom; 4 Aneurin Bevan University Health Board Cardiff, Wales United Kingdom

**Keywords:** breast cancer diagnosis, artificial intelligence, AI adoption, barriers and facilitators, mixed methods, physician perceptions, innovation, technology acceptance model, Austria, early detection

## Abstract

**Background:**

Artificial intelligence (AI) has demonstrated strong potential in breast cancer diagnostics by improving accuracy, efficiency, and clinical workflow. However, adoption among physicians remains variable. Existing research often overlooks the contextual and experiential differences between clinicians who use AI and those who do not. A comprehensive understanding of barriers and facilitators, especially across user groups, is essential to inform equitable and effective AI implementation in real-world settings.

**Objective:**

This study aimed to (1) identify key barriers and facilitators influencing the use of AI tools in breast cancer diagnostics, with a specific focus on comparing current users and nonusers, and (2) examine how social, technological, and individual-level factors are linked to physicians’ attitudes toward AI, intention to use it, and perceived likelihood of future adoption.

**Methods:**

A cross-sectional, embedded mixed methods survey was conducted with 46 Austrian physicians. Quantitative items were based on the technology acceptance model and its extensions. Open-ended responses were analyzed using conventional content analysis and integrated with quantitative results via joint displays. Ordinary least squares regressions examined factors associated with attitudes, intention, and the likelihood of future AI use.

**Results:**

Among the 46 participating physicians, 52% (n=24) reported current AI use. Common facilitators included improved quality of work, efficiency, and expanding knowledge. Nonusers highlighted barriers such as limited access (17/21, 81%), high costs, and lack of training. AI users highlighted barriers related to limited integration with existing systems and concerns about trust. Despite these differences, both groups expressed strong future adoption intentions. Perceiving multiple facilitators was significantly associated with more favorable attitudes (B=0.83; *P*=.02), stronger intention to use AI (B=1.32; *P*=.01), and higher perceived likelihood of future use (B=1.56; *P*=.001). AI-related skills positively predicted intention (B=1.00; *P*=.04) and likelihood of future use (B=1.16; *P*=.01), while colleagues’ positive views about AI predicted both attitudes (B=0.34; *P*=.02) and intention (B=0.39; *P*=.01). In contrast, perceiving multiple barriers was associated with lower intention (B=–0.84; *P*=.047) and likelihood (B=–1.48; *P*<.001). Being aged 50 or older was significantly associated with more negative attitudes (B=–1.11; *P*=.002) and lower likelihood of future use (B=–0.82; *P*=.02).

**Conclusions:**

This study offers preliminary insights into the implementation of AI in breast cancer diagnostics within the Austrian health care context. AI adoption appears to be a staged process with evolving support needs. Early-stage users may benefit from improved access and training, while experienced users require support for workflow integration and trust-building. Promoting peer support, addressing demographic disparities, and embedding AI training into clinical routines may support more sustainable and equitable adoption. These findings inform tailored implementation strategies and offer recommendations that may be transferable to other health systems.

## Introduction

### Background

Breast cancer is the most common cancer among women globally and a leading cause of cancer-related morbidity and mortality [1,2]. Early and accurate diagnosis is essential to improving outcomes, yet remains vulnerable to human error, including interpretive variability and diagnostic inaccuracies [3]. In Austria, nearly 90,000 women were living with breast cancer as of 2022 [4], and increasing incidence projections combined with physician shortages underscore the need for scalable and efficient diagnostic tools [5,6].

Artificial intelligence (AI) systems trained on large datasets have demonstrated strong performance in detecting malignancies and reducing diagnostic errors [7,8]. In some screening contexts, AI has even outperformed radiologists in stand-alone mammography detection tasks [9]. Nevertheless, AI is not intended to replace clinicians, and concerns remain regarding overreliance on automated systems without appropriate human oversight, which could have serious clinical consequences [10]. Accordingly, AI is increasingly conceptualized as a decision-support tool that may enhance diagnostic efficiency, accuracy, and workflow while preserving clinician responsibility [11-13].

Despite its potential, the adoption of AI in clinical practice remains uneven across health care systems [5]. International research consistently shows that integration into routine care is hindered by a combination of organizational, technical, and human factors [14,15]. Understanding barriers and facilitators to AI adoption is therefore critical for enabling ethical, effective, and sustainable implementation [14].

### Theoretical Framework

The technology acceptance model (TAM) posits that technology uptake is influenced by perceived usefulness (ie, the expected benefits of use) and perceived ease of use (ie, the anticipated effort required), which together inform users’ attitudes and subsequently influence behavioral intention and actual use [16,17]

Extensions of TAM, including TAM2, incorporate subjective norms, capturing the influence of perceived social pressure on adoption decisions [18,19]. The Unified Theory of Acceptance and Use of Technology (UTAUT) further integrates these concepts by identifying by identifying performance expectancy (expected benefits), effort expectancy (expected ease of use), social influence (influence of important others), and facilitating conditions (perceived organizational and technical support) as core determinants of technology acceptance, moderated by individual characteristics such as age, sex, and experience [19].

Across international health technology research, perceived usefulness, usability, and social norms have been consistently associated with clinicians’ willingness to adopt digital innovations [14,20,21]. In radiology and oncology settings, perceived benefits such as improved diagnostic accuracy, workflow efficiency, and enhanced quality of patient care are commonly reported facilitators [22-24]. Further health research found that systems perceived as intuitive and well-integrated into clinical workflows are more likely to be adopted, highlighting the importance of design usability and operational fit [21]. Conversely, persistent barriers include limited infrastructure, high costs, lack of training, workflow disruption, and concerns about increased workload [25].

Qualitative evidence further highlights psychological and professional concerns, including limited AI knowledge, lack of explainability, perceived threats to professional autonomy, and fears of role displacement [26]. In radiology in particular, unfamiliarity with AI tools and concerns about reduced autonomy have been repeatedly identified as salient barriers [21]. Beyond traditional TAM and UTAUT constructs, trust has emerged as a critical determinant of AI adoption [27]. Research suggests that system transparency, reliability, and explainability are integral to building clinician trust, which in turn significantly influences the likelihood of adopting AI-based tools [27].

While AI acceptance has been explored in general health care contexts, its specific application in breast cancer diagnostics remains underexamined, despite growing clinical relevance [9]. Country-specific factors, including regulatory frameworks, digital infrastructure, and workforce capacity, are likely impacting the health care system’s readiness to adopt AI [28]. More targeted research is therefore needed to understand the factors influencing adoption, particularly within national contexts such as Austria [21].

In addition, international studies have identified a range of barriers and facilitators to AI use and suggest that attitudes may vary by level of AI experience [21,26]. However, few studies have directly compared clinicians who currently use AI with those who do not, limiting insight into how hands-on AI experience influences attitudes, perceived barriers, and implementation readiness.

### Objectives

To address the aforementioned gaps, this study investigates AI use in breast cancer diagnostics within the Austrian health care system and explicitly compares physicians with and without direct AI experience.

Specifically, this study has two primary objectives:

1. To identify key barriers and facilitators influencing the adoption of AI for breast cancer diagnostics among Austrian physicians and to compare these across current AI users and nonusers.
2. To examine how technology-related factors (eg, perceived usefulness, ease of use), social factors (eg, peer influence), and individual characteristics (eg, age, sex, AI-related skills) are associated with physicians’ attitudes toward AI, behavioral intentions, and perceived likelihood of future AI adoption.

These insights aim to inform practical, evidence-informed, and human-centered strategies for integrating AI into breast cancer diagnostics [29].

## Methods

### Study Design

We used an embedded mixed methods [30], cross-sectional questionnaire design, adopting a pragmatic epistemological stance [31] to prioritize methodological fit with the study aims. The study examined (1) barriers and facilitators to AI adoption among AI users and nonusers using quantitative descriptive analyses complemented by qualitative open-ended responses, and (2) predictors of attitudes, intentions, and perceived likelihood of future AI use using quantitative regression analyses.

### Recruitment and Data Collection

This study was conducted as part of a master’s thesis project at the University for Continuing Education Krems, Austria (EH). Eligible participants were practicing physicians involved in breast cancer screening or diagnostics. Exclusion criteria included nonphysicians, retired clinicians, and those not actively engaged in diagnostic work.

Data collection took place over an 8-week period from March to April 2023. A German-language online survey was distributed via predefined professional and institutional channels using email invitations and an electronic flyer. Primary dissemination occurred through the Austrian Medical Chamber (Ärztekammer), complemented by distribution via university channels, social media platforms, and relevant professional networks. Participants accessed the survey via a secure link, and only respondents who consented and met the predefined screening criteria were able to proceed with the survey.

### Ethical Considerations

The study received ethical approval from the Ethics Committee of the University for Continuing Education Krems (EKGZ 55/2021-2024). All participants provided informed consent electronically before accessing the survey. Participation was voluntary, and respondents could withdraw at any time without consequences. Data were collected anonymously, with no personally identifiable information recorded. Secure data storage procedures were applied to protect confidentiality. No financial compensation was provided for participation.

### Measures

Quantitative survey items were adapted from previously validated TAM [16,20] instruments and informed by relevant digital health research [21]. Items were tailored to the context of breast cancer diagnostics [16,19]. The first section captured sociodemographic data, AI awareness, and usage. Sex was assessed via self-report; sex or sex assigned at birth was not collected. Attitudes toward AI were measured using two 7-point Likert items averaged into a composite score; additional items included perceived AI-related skills, perceived usefulness, and perceived attitudes of colleagues. The second section addressed perceived barriers (eg, limited availability, high costs, usability, data concerns) and facilitators (eg, efficiency, diagnostic precision, stress reduction) using multiple-choice and open-ended items. The final section measured future intention and likelihood of AI use with a single 7-point Likert item, followed by optional free-text elaborations. Item phrasing was adapted to reflect whether participants were current AI users or nonusers.

Qualitative data were collected through open-ended questions embedded in the online survey, which invited participants to elaborate on perceived barriers and facilitators to the use of AI in breast cancer diagnostics. Data collection was limited to the survey format, and no additional qualitative follow-up (eg, interviews) was conducted. The full wording of all the translated survey items is provided in Multimedia Appendix 1.

### Data Analysis

#### Quantitative Data Analysis

All quantitative analyses were performed using Stata (version 18; StataCorp LLC, College Station, TX, USA). Descriptive statistics summarized participant characteristics and key variables, with means and SDs reported for continuous variables, and frequencies and percentages for categorical variables. Between-group comparisons of perceived barriers and facilitators (users vs nonusers) were conducted using Mann–Whitney U tests (α=0.05) due to nonnormal distributions.

To examine factors associated with attitudes, intentions, and the likelihood of future AI use, multiple linear regression analyses (ordinary least squares) were conducted.

Model assumptions were formally assessed using variance inflation factors for multicollinearity, Shapiro–Wilk tests and Q–Q plots for normality of residuals, and Breusch–Pagan and White tests for heteroskedasticity. All regressions were estimated with heteroskedasticity-robust (Huber–White) standard errors. Sensitivity analyses using ordered logistic regression were conducted to account for the ordinal nature of the outcomes. The detailed results of these diagnostics are reported in Multimedia Appendix 2.

Predictors included sociodemographic variables (eg, age and sex), current AI use, perceived usefulness, AI skills, and colleagues’ attitudes. Barriers and facilitators were coded as binary variables. To assess robustness, alternative codings were also tested, including any/none dummy coding and continuous sum scores. Results of these sensitivity analyses are reported in the Multimedia Appendix 3. Missing data were minimal, and no imputation procedures were performed. Analyses were conducted using available case data for each analysis. The number of observations included is reported where relevant.

#### Qualitative Data Analysis

Open-ended survey responses were analyzed using conventional content analysis [32] following an inductive, iterative process. The first author (MK), who has experience in qualitative and mixed methods research, conducted open coding by assigning descriptive labels to meaningful text segments to capture participants’ perspectives. Codes were generated directly from the data and iteratively refined.

Related codes were then grouped into broader categories based on recurring patterns. In a second step, categories were deductively organized into 2 overarching domains, barriers and facilitators, and aligned with the structure of the quantitative survey, consistent with the study’s embedded mixed methods design. This enabled qualitative findings to elaborate on and contextualize quantitative results. For example, while a closed-ended item identified “expanding knowledge” as a facilitator, the qualitative category “AI as a Catalyst for Continuous Learning and Professional Growth” provided additional insights, describing AI’s role as a training tool and support

To strengthen analytic rigor and reflexivity, the developing coding structure and category definitions were reviewed and discussed with a second researcher (WH). These discussions supported critical reflection and refinement of interpretations. During analysis, the emergence of new categories was monitored, and no substantively new categories were identified in later responses, indicating informational redundancy in the open-ended data [32]. Additional coding details and illustrative quotes are provided in Multimedia Appendix 3.

#### Data Integration

To address the first research aim, we used joint displays to integrate qualitative and quantitative findings. Two integrated tables, one for barriers and one for facilitators, summarized corresponding categories, representative codes, and relevant quantitative frequencies. This side-by-side comparison enabled us to identify points of convergence and divergence between AI users and nonusers and provided contextual insight that helped explain and expand upon the statistical results [31,33]. The second research aim was addressed using quantitative analyses only. Findings from both research aims and from both qualitative and quantitative strands, were synthesized in the Discussion to generate context-specific and actionable recommendations for supporting AI integration in clinical settings.

## Results

### Descriptive Statistics

A total of 53 participants completed the survey. Seven participants were excluded: 6 participants because they were not currently working in breast cancer diagnostics and one participant due to missing demographic and professional information. The final sample comprised 46 participants. Of these, most (40/46, 87%) reported being aware of AI technologies for breast cancer diagnosis, and approximately half (24/46, 52%) indicated they currently use AI in their diagnostic practice. The sociodemographic characteristics of the final sample are summarized in Table 1.

**Table 1:** Sociodemographic characteristics of the participants (N=46).

Characteristic		Values
**Age (years) (n=45)^a^**		
	Mean (SD)	53.8 (8.64)
	Range	37-72
**Sex**		
	Female, n (%)	11 (23.9)
**Region (n=44)^b^, n (%)**		
	Vienna	32 (72.7)
	Lower Austria	4 (8.5)
	Styria	4 (8.5)
	Upper Austria	2 (4.3)
	Corinthia	2 (4.3)
**Current position, n (%)**		
	Self-employed	18 (39.1)
	Physician in training (hospital)	2 (4.4)
	Senior physician (hospital)	15 (32.6)
	Department head (hospital)	5 (10.9)
	Other	8 (17.4)
**Field of specialization, n (%)**		
	Radiologist	17 (37)
	Gynecologist	9 (19.6)
	Internal medicine	3 (6.5)
	Oncology	3 (6.5)
	Pathology	3 (6.5)
	Surgery	2 (4.4)
	Rheumatology	1 (2.2)
	Other	8 (17.4)
**Tasks, n (%)^c^**		
	Mammography	28 (60.9)
	Ultrasound	26 (56.5)
	Magnetic Resonance Imaging (MRI)	21 (45.6)
	Computed Tomography (CT)	19 (41.3)
	Dual-Energy X-ray Absorptiometry (DXA)	15 (32.6)
	Positron Emission Tomography (PET)	6 (13)
	Angiography/Fluoroscopy	4 (8.7)
	Hybrid imaging	3 (6.5)
	Experimental imaging	2 (4.3)
	Other	13 (28.3)

^a^Age data were missing for 1 participant.

^b^Region data were missing for 2 participants.

^c^Multiple responses possible.

### Barriers and Facilitators to AI Use

Responses to barrier items were available for 44 participants, and responses to facilitator items for 41 participants. Qualitative data were derived from open-ended responses provided by 41 physicians (56% AI users). The qualitative analysis yielded 21 codes, comprising 12 facilitator categories and 9 barrier categories related to AI adoption. An integrated overview of qualitative categories and corresponding quantitative frequencies is presented in Tables 2 and 3. Additional details on qualitative categories, codes with illustrative quotes, and aligned quantitative data are provided in Multimedia Appendix 3.

**Table 2:** An integrated presentation of mixed methods findings on perceived barriers to artificial intelligence adoption among physicians. Quantitative item labels were shortened for table presentation; full questionnaire items are provided in Multimedia Appendix 1. Qualitative codes and illustrative quotes corresponding to each category are provided in Multimedia Appendix 3. Multiple barriers could be endorsed by each participant. There was a statistically significant difference between artificial intelligence users and artificial intelligence nonusers (z=3.879; *P*<.001).

Quantitative item	Overall (N=44), n (%)	AI^a^ users (n=23), n (%)	AI nonusers (n=21), n (%)			Qualitative category
High costs	17 (39)	8 (35)		9 (43)	Access, cost, and integration challenges	
Not available at the workplace	22 (50)	5 (22)		17 (81)	Access, cost, and integration challenges	
Difficult technical integration into existing systems	14 (32)	9 (39)		5 (24)	Access, cost, and integration challenges	
Lack of user-friendliness or operability	12 (27)	8 (35)		4 (19)	Usability and training needs	
Lack of knowledge to use AI for breast cancer diagnostics	11 (25)	4 (17)		7 (33)	Usability and training needs	
IT^b^ security concerns	8 (18)	6 (26)		2 (10)	Data privacy and IT security concerns	
Data protection concerns	6 (14)	4 (17)		2 (10)	Data privacy and IT security concerns	
Lack of effectiveness	5 (11)	3 (13)		2 (10)	Workflow disruption and limited trust	
Workflow incompatibility or limited trust in AI	5 (11)	3 (13)		2 (10)	Workflow disruption and limited trust	

^a^AI: artificial intelligence.

^b^IT: information technology.

**Table 3:** An integrated presentation of mixed methods findings on perceived benefits of artificial intelligence adoption among physicians. Quantitative item labels were shortened for table presentation; full questionnaire items are provided in Multimedia Appendix 1. Qualitative codes and illustrative quotes corresponding to each category are provided in Multimedia Appendix 3. Multiple benefits could be endorsed by each participant. There was a statistically significant difference between users and nonusers (z=−2.19; *P*=.03).

Quantitative item	Overall (N=41), n (%)	AI^a^ users (n=23), n (%)	AI nonusers (n=18), n (%)	Qualitative category
More accurate diagnostics	26 (63)	14 (61)	12 (67)	Improving diagnostic confidence and trust in accuracy
Quality improvement of work through better patient care	17 (41)	13 (57)	4 (22)	Improving diagnostic confidence and trust in accuracy
Faster task completion	16 (39)	8 (35)	8 (44)	Improved efficiency as a catalyst for enhanced quality of care
Simplifying work processes	8 (20)	4 (17)	4 (22)	Reducing stress and enhancing well-being
Feeling less stressed	6 (15)	3 (13)	3 (17)	Reducing stress and enhancing well-being
More control over workflow	3 (7)	1 (4)	2 (11)	Reducing stress and enhancing well-being

^a^AI: artificial intelligence.

### Barriers to AI Adoption

Among participants who completed the barrier items (n=44), most (40/44) reported at least one perceived barrier to AI use. Conventional content analysis identified 9 codes pertaining to barriers, which were organized into 4 overarching categories. Table 2 presents a joint display of perceived barriers to AI adoption, integrating quantitative response frequencies with qualitative categories and codes.

### Access, Cost, and Integration Challenges

Infrastructural, financial, and technical limitations were among the most frequently cited barriers to AI adoption. Availability was reported as a significant concern, particularly among nonusers: 81% (17/21) of nonusers indicated that AI tools were not accessible in their workplace, compared with only 22% (5/23) of users (*P*<.001; z=3.879). Qualitative responses elaborated on these findings, with nonusers noting, “The software is not available in the department,” or “AI tools are not yet integrated in my workplace.” High costs were also raised as a concern, reported by 43% (9/21) of nonusers and 35% (8/23) of users, though this difference was not statistically significant. Several participants described AI as being “too costly to implement here,” underscoring the perceived financial barriers in resource-constrained environments.

In addition to cost, technical integration was reported as a key limitation by 32% (14/44) of participants, with significantly higher prevalence among users (9/23, 39%) compared with nonusers (5/21, 24%). Qualitative accounts contextualized these concerns: several AI users noted that current systems were not fully compatible with existing software, adding rather than reducing workflow burden. As one user explained, “AI doesn’t align well with our current software and therefore adds to the workflow.”

When asked about motives for future use, several participants emphasized that seamless integration and workplace availability are essential preconditions for future use. As one nonuser noted, “AI must be readily available and smoothly integrated to be adopted in daily practice”. Collectively, these results highlight how cost, resource access, and system compatibility may act as gatekeepers that must be addressed to enable broader and sustained AI adoption in clinical settings.

### Usability and Training Needs

Both quantitative and qualitative data indicated that access to training and ease of use are critical factors influencing AI adoption. Among current users, 35% (8/23) indicated limited user-friendliness as a challenge. In contrast, 33% (7/21) of nonusers reported a lack of knowledge with AI as a barrier. Qualitative responses provided additional context. One nonuser noted, *“*I haven’t used AI because it’s still a new concept and training hasn’t been offered*,”* highlighting the absence of structured onboarding opportunities. This category was also reinforced with responses to the question about what would motivate future use: nonusers, in particular, emphasized the need for training to build confidence and capability.

### Data Privacy and IT Security Concerns

Among AI users, concerns about IT security (26% vs 10%) and data protection (17% vs 10%) were slightly more prominent compared with nonusers, though these differences were not statistically significant. Qualitative responses supported these findings, with 3 AI users raising concerns about patient privacy and data security. For example, one respondent noted, “AI brings up data protection issues for patients”, suggesting that security considerations may become increasingly important as AI use expands.

### Workflow Disruption and Limited Trust

Challenges related to workflow disruption and trust in AI outputs were primarily reported by current AI users. Although AI was generally perceived to enhance productivity, 11% (5/44) of participants reported a lack of effectiveness as a barrier. This concern was elaborated upon in open-ended responses, which described how AI systems occasionally generated false alarms or required additional verification steps, thereby limiting potential time savings. For example, one user explained, “AI sometimes creates extra work due to false alarms”, highlighting the potential for AI to unintentionally increase workload.

Trust in AI outputs also emerged as an important issue. Several participants described a need to double-check AI-generated results. One user stated, “I trust AI, but I double-check its suggestions”, suggesting that partial trust may reduce the efficiency benefits of AI by necessitating manual oversight. These findings indicate that both technical and psychological barriers, specifically, limited system integration and cautious trust, may attenuate the intended benefits of AI in clinical workflows.

### Facilitators to AI Adoption

#### Overview

Descriptive statistics showed that all AI users (n=23) and most nonusers (16/18) reported at least one perceived benefit of AI. Conventional content analysis identified 10 codes, which were organized into 4 overarching facilitator-related categories. Table 3 provides an integrated overview of perceived facilitators to AI adoption in breast cancer diagnostics to AI adoption, integrating quantitative response frequencies with qualitative categories and codes.

#### Improving Diagnostic Confidence and Trust in Accuracy

The most frequently endorsed facilitator was the perception that AI improves diagnostic accuracy, reported by 63% (26/41) of participants. Both users and nonusers acknowledged this benefit, suggesting its value extends beyond direct experience. Among users, AI was commonly described as a reliable second opinion that enhanced certainty in complex cases by improving diagnostic accuracy and reducing the risk of misdiagnosis. As one physician noted, “AI provides an extra layer of security in diagnostics,” illustrating how AI can support confidence in clinical decision-making and contribute to the overall quality of diagnostic work. When asked about future use, physicians mentioned that trust in AI accuracy would need to be further strengthened through “guideline recommendations and scientific evidence,” which was considered a motivator for future adoption. This highlights the importance of scientific validation for reinforcing diagnostic trust as a condition for sustained or future use.

#### Improved Efficiency as a Catalyst for Enhanced Quality of Care

This category pertains to how AI was perceived as streamlining routine diagnostic tasks, increasing productivity, and enabling clinicians to focus more on patient-centered care and complex decision-making. The link between efficiency gains and improved care quality emerged consistently across qualitative and quantitative responses. Improved efficiency was cited by over one-third of participants. One nonuser explained, “It could reduce the processing time per patient and enable more timely diagnoses*.*”

Similarly, 2 out of 18 nonusers and 1 out of 23 users identified improved workflow as a key advantage. Qualitative responses highlighted that AI integration was often seamless; as one AI user elaborated: “The use is integrated into the software and is not consciously perceived*.*” Improved quality of care was identified by 41% (17/41) of respondents, with users (13/23, 57%) significantly more likely than nonusers (4/18, 22%) to emphasize this benefit (z=–2.185; *P*=.03).

Several AI users commented that AI’s efficiency in handling specific tasks allowed them to focus on other responsibilities. One physician stated, “AI lets me focus on patients rather than routine diagnostics,” while another noted, “AI saves time on diagnostics, allowing me to focus on complex cases.” These findings collectively suggest that AI not only expedited routine processes but also enabled clinicians to concentrate on higher-value tasks, enhancing both productivity and patient care.

#### AI as a Catalyst for Continuous Learning and Professional Growth

Quantitative results indicated that over one-third of participants (39%) identified AI as a valuable learning tool, with both users and nonusers attributing equal importance to its role in professional development. Qualitative findings supported this view, revealing that AI was perceived as beneficial for both self-directed learning and the training of junior staff. As one participant noted, *“*As a training tool, AI transforms routine diagnostics into teachable moments,*”* highlighting AI’s potential to facilitate on-the-job learning and support ongoing clinical education.

#### Reducing Stress and Enhancing Well-Being

This category summarizes how AI was perceived to alleviate workload and mitigate stress, offering psychological benefits. Both users and nonusers identified simplifying work (8/41, 20%) and stress reduction (6/41, 15%) as key benefits. For example, one user noted, “AI decreases the pressure on me during busy shifts*,”* suggesting that AI contributes to a more balanced and manageable work environment. Looking forward, participants suggested that future adoption would depend on clear cost-benefit ratios and minimal added complexity. As one respondent put it, “AI will be used more if it delivers measurable improvements without unnecessary complications.” This may suggest that emotional and logistical facilitators, such as reduced stress and usability, are contingent on thoughtful system design and implementation.

### Summary of Barriers and Facilitators

High costs, limited availability, and training needs were identified by both users and nonusers as key barriers to AI adoption. Nonusers more frequently cited access and capability-related challenges, whereas users highlighted issues related to system integration and workflow disruptions as barriers.

Across both groups, improved diagnostic accuracy emerged as the most frequently mentioned facilitator of AI use in breast cancer diagnostics. Users emphasized several clinical benefits, including improved patient care and increased efficiency. Nonusers also acknowledged AI’s potential to support efficiency but placed greater emphasis on the importance of enabling conditions, such as structured training and workplace accessibility, to support future adoption. Addressing both shared and group-specific factors will be essential for developing targeted implementation strategies and ensuring broader, equitable uptake of AI in clinical practice.

### Factors Determining Attitude, Intention, and Perceived Likelihood of Future Use

#### Overview

To address the second research aim, ordinary least squares regression analyses were conducted to identify factors associated with physicians’ attitudes, intentions, and perceived likelihood of future AI use. Complete regression data were available for 41 respondents. Detailed descriptive statistics and robustness analyses are reported in Multimedia Appendix 2.

#### Attitudes Toward AI

The regression results are presented in Table 4 and indicate that physicians aged 50 or older held significantly less favorable attitudes toward AI (B=–1.111; *P*=.002), possibly reflecting generational differences in technological exposure or comfort with AI integration. Perceived social norms also emerged as influential: physicians who reported that their colleagues consider AI important report a significantly more positive attitude (B=0.341; *P*=.02). Additionally, perceiving at least 2 facilitators of AI was significantly associated with a more favorable attitude (B=0.832; *P*=.02), emphasizing the importance of perceived advantages in driving AI acceptance. Conversely, although the association did not reach statistical significance (*P*=.07), perceiving 2 or more barriers to AI adoption was associated with a trend toward less favorable attitudes (B=–0.501, SE=0.268), indicating that concerns about risks, implementation challenges, or ethical issues may influence clinicians’ views. Overall, the model accounted for 62.2% of the variance in attitudes (*R*^2^=0.622).

**Table 4:** Ordinary least squares regression results for factors influencing attitudes, intentions, and perceived likelihood of future artificial intelligence use (N=41).

Independent variables	Attitude toward AI^a^ use (*R*^2^=0.62)			Intention to use AI (*R*^2^=0.65)			Likelihood of future AI use (*R*^2^=0.70)	
	B (SE)^b^	*P* value	B (SE)^b^		*P* value	B (SE)^b^		*P* value
Facilitators	0.83 (0.33)	.02	1.32 (0.51)		.01	1.56 (0.42)		<.001
Barriers	−0.50 (0.27)	.07	−0.84 (0.41)		.047	−1.48 (0.37)		<.001
AI user	−0.02 (0.29)	.94	0.66 (0.47)		.17	1.29 (0.47)		.01
Skills for using AI	0.38 (0.32)	.24	1.00 (0.47)		.04	1.16 (0.43)		.01
Colleagues consider AI important	0.34 (0.14)	.02	0.39 (0.14)		.01	−0.03 (0.18)		.87
Colleagues’ opinion is important	0.05 (0.08)	.55	−0.04 (0.08)		.66	0.06 (0.10)		.57
Female	−0.24 (0.37)	.52	0.01 (0.42)		.981	−0.86 (0.47)		.08
Age ≥50 years	−1.11 (0.32)	.002	−0.81 (0.40)		.053	−0.82 (0.34)		.02
Constant	4.03 (0.76)	<.001	2.94 (0.83)		.001	4.25 (0.70)		<.001

^a^AI: artificial intelligence.

^b^B indicates regression coefficient; robust standard errors are shown in parentheses.

#### Intention to Use AI for Breast Cancer Diagnosis in the Future

It was found that physicians who perceived more benefits (*P*=.01) and fewer barriers (*P*=.047) to AI reported a higher intention to adopt it in their practice. This highlights the crucial role of both perceived advantages and reduced obstacles in shaping the willingness to integrate AI into clinical decision-making. Furthermore, physicians who perceived that their colleagues considered AI use important were more likely to express an intention to use AI themselves, underscoring the influence of social norms in professional environments.

Younger physicians demonstrated a higher intention to adopt AI, which, similarly to attitudes, may reflect greater exposure to technological advancements and a higher level of adaptability to digital innovations in health care. Additionally, possessing skills related to AI use was positively associated with the intention to adopt it, reinforcing the importance of competence and confidence in facilitating future implementation. The model explained 64.7% of the variance (*R*^2^=0.647). Taken together, these results underscore the combined influence of perceived benefits, social context, and capability beliefs in influencing physicians’ intentions to use AI in the future.

#### Perceived Likelihood of Future AI Use

The regression analysis for the perceived likelihood of using AI in the future is presented in Table 4. Consistent with the models for attitudes and intention to use AI, perceiving facilitators was positively associated with the likelihood of future AI use, while perceived barriers were negatively associated.

Both current AI use and possessing AI-related skills were statistically significant predictors of the likelihood of using AI in the future for breast cancer diagnostics. While current AI use was not significantly related to attitudes or intentions, it was a significant predictor of perceived likelihood, suggesting that actual use may determine future expectations more than current attitudes.

Physicians aged 50 years and above consistently demonstrated a lower likelihood of future adoption across all 3 models, reaffirming the key role of age in shaping expectations around AI use in clinical practice. In contrast, gender was not a statistically significant predictor in any of the models. For the likelihood of future AI use, the coefficient for female physicians was negative (B=–0.856; *P*=.08), which is not significant at the conventional α of 0.05 level but would be considered marginally significant at a liberal threshold of *P*≤.1.

These findings were robust across alternative model specifications. Facilitator and barrier variables were re-coded using binary indicators (≥1 vs 0) and continuous sum scores, with consistent results. Additional robustness checks using ordered logistic regression and reduced models including only key predictors yielded comparable estimates. Full regression diagnostics and sensitivity analyses are reported in Tables S2-S5 in Multimedia Appendix 2.

## Discussion

### Principal Findings

This study aimed to examine the key barriers and facilitators to AI for breast cancer diagnosis among Austrian physicians, with a specific focus on comparing current users and nonusers. We also sought to identify how individual, social, and technological factors predict physicians’ attitudes toward AI, their intention to use it, and their perceived likelihood of future adoption. While most participants were aware of AI technologies, only approximately half had used such technologies in their clinical practice.

Key facilitators included perceiving benefits in terms of diagnostic accuracy, professional growth, workflow efficiency, and overall quality of patient care. Barriers included limited access, high costs, integration issues, and insufficient training opportunities. Nonusers reported anticipated benefits, such as improved diagnostic accuracy and workflow efficiency, but identified limited availability as a key barrier to adoption. In contrast, users cited concrete benefits, including enhanced diagnostic precision, quality of care, and productivity, while also expressing some concerns regarding cost, integration with existing systems, and trust in AI-generated outputs.

Regression analyses indicated that perceptions of more facilitators and fewer barriers were strongly associated with more favorable attitudes and higher adoption intentions. Social and demographic factors also played a role: younger physicians and those in environments where colleagues valued AI were more likely to have favorable attitudes and stronger intentions to use AI. Experience with AI and related skills were consistent predictors of intention and perceived likelihood of future use, while older age was negatively associated with adoption likelihood.

### Comparison With Prior Work and Theoretical Implications

#### Awareness and Use of AI

Our findings align with previous work demonstrating moderate levels of AI use among clinicians. Approximately half of our sample reported current AI use, comparable to usage rates found by Shinners et al [34] and Zanardo et al [35], who noted usage rates of 50% and 48%, respectively. In contrast, Chen et al [5] found a usage rate of 72%, possibly due to their sample being composed of resident radiologists. This aligns with prior work suggesting that physicians in training may exhibit higher readiness to use AI [34]. The slightly higher unawareness rate in our study (13%) compared with other specialties (eg, 4.3% in Chen et al [5]) may reflect our focus on breast cancer diagnostics rather than general radiology.

#### Barriers and Facilitators to AI Use

Consistent with prior international research, improved diagnostic accuracy and workflow efficiency emerged as the most frequently cited facilitators of AI adoption [20,21,24]. Theoretically, these results align with the UTAUT, particularly the construct of performance expectancy as a key determinant of behavioral intention [19]. Studies among radiologists and oncologists in Europe and Asia similarly identify performance expectancy as a key driver of favorable attitudes toward AI [22,35]. However, our findings indicate that these perceived benefits are also widely acknowledged by clinicians without direct AI experience, suggesting that positive attitudes alone are insufficient to prompt adoption in the absence of appropriate structural support.

In this context, the prominence of structural and organizational barriers among nonusers becomes particularly salient. Limited access, costs, and training deficits, reported primarily by clinicians without AI experience, align with international literature identifying resource constraints as critical barriers to digital health implementation [14,25]. These findings align with UTAUT’s emphasis on facilitating conditions, such as infrastructure, institutional support, and technical resources, as key precursors of technology use [19]. This pattern is also consistent with recent CFIR-guided evidence syntheses, which highlight that safe and sustainable AI adoption in breast screening requires attention not only to perceived benefits, but also to technological integration, training, trust, and governance [15].

The strong emphasis on training needs, particularly among nonusers, aligns with the UTAUT construct of effort expectancy and TAM’s notion of perceived ease of use, both of which highlight the importance of minimizing the perceived complexity of new technologies [16,19]. The finding that nonusers expressed willingness to adopt AI if training were available suggests that lower perceived effort through structured onboarding may act as a critical enabler. This finding reinforces the need for user-friendly systems and accessible, targeted training programs, especially for clinicians with limited prior exposure to AI.

The finding that AI users raised concerns related to workflow integration and trust in outputs is consistent with other studies demonstrating that real-world adoption surfaces unique operational and cognitive demands [26]. Notably, lack of trust, especially regarding the accuracy and reliability of AI outcomes, has been widely identified as a barrier to adoption [27,36]. These concerns highlight trust as a central determinant that interacts with, and potentially moderates, traditional TAM/UTAUT constructs, aligning with growing calls to integrate explainability and transparency into health technology acceptance frameworks [15,27]. In our study, participants also highlighted that scientific guidelines could enhance trust in AI outputs, particularly among those who were hesitant to rely on algorithmic support without established clinical validation. Taken together, the contrast between users and nonusers underscores that AI adoption may not be a binary decision but a staged process, in which anticipated barriers dominate early adoption phases, while experiential challenges emerge with sustained use.

#### Influences on Attitudes, Intentions, and Likelihood of Future AI Use

While prior work has focused on attitudes as the primary driver of behavioral intention [5], our findings emphasize the additive role of perceived barriers and facilitators. A greater number of perceived barriers corresponded with lower adoption intentions, while more perceived benefits predicted stronger intentions. These results underscore the need for a dual strategy: promoting perceived advantages while actively addressing barriers.

Peer perceptions also played an important role. Physicians who perceived that AI was valued by their colleagues were more likely to report positive attitudes and stronger intentions to adopt the technology. The finding that perceiving benefits was linked to intention and the role of subjective norms is consistent with TAM 2 and UTAUT, which both emphasize perceived usefulness and social influence as key determinants of adoption behavior [18,19]. Peer endorsement may support adoption, particularly among clinicians with limited AI experience, consistent with recommendations to incorporate social and organizational influences, such as colleague support, into models of technology uptake in health care [20].

Age was a consistent negative predictor across all models, with older physicians reporting significantly lower attitudes, intention, and likelihood of future AI adoption. This may reflect generational differences in technology familiarity, perceived disruption to routine, or risk tolerance [5,27]. Conversely, prior experience with AI in clinical practice was positively associated with future intention to use, aligning with earlier findings that experience and exposure enhance acceptance [5,37].

Taken together, these findings extend international evidence by demonstrating that differences between AI users and nonusers are not merely attitudinal but reflect distinct stages of implementation readiness, with implications for how AI adoption strategies should be tailored across clinical contexts. Recognizing this progression may inform more targeted and flexible strategies. However, these patterns are preliminary and hypothesis-generating and should be confirmed in larger samples.

### Practical Implications

Practically, our findings offer valuable insights for health care leaders, educators, and policymakers aiming to support the responsible integration of AI in breast cancer diagnostics. Implementation strategies could benefit from not only highlighting perceived advantages but also addressing specific barriers that appear to differ between current users and nonusers.

As outlined in Table 5, potential strategies may include consideration of demographic differences (eg, age), social norms, and levels of technical readiness. Targeted support, such as age-appropriate training, peer-led initiatives, and structured upskilling opportunities, might help reduce disparities in AI engagement. Findings suggest that adoption may not be a binary decision but a staged process with evolving support needs. Early-stage users may benefit from structural interventions (eg, access and training), while more experienced users may require support with integration and workflow adaptation. This distinction implies that implementation efforts may need to be adapted over time and tailored to users’ varying levels of familiarity and experience with AI.

**Table 5:** Summary of the recommendations based on key findings for artificial intelligence use in health care.

Factor	Key finding	Recommendation
Demographic factors	Older age was associated with less favorable attitudes toward AI^a^ and lower intention and likelihood of future use.	Develop age-responsive training and support strategies to address barriers experienced by older clinicians.
Barriers	Perceived barriers were negatively associated with attitudes, intention, and likelihood of AI use.	Address identified barriers through targeted education, technical support, and organizational resources to improve attitudes and facilitate adoption.
Facilitators	Perceived facilitators were positively associated with attitudes, intention, and likelihood of AI use.	Promote success stories and demonstrated benefits of AI to strengthen positive perceptions and encourage adoption.
Knowledge and use of AI	Greater AI-related skills were associated with higher intention and likelihood of future use, but not with attitudes. Current AI users were more likely to report future use.	Invest in practical AI training programs to build skills and confidence. Support experienced users to act as champions and mentors for wider adoption.
Social factors	Colleagues’ perception of AI’s importance positively influenced attitude and intention, but not the likelihood of future AI use.	Foster a supportive workplace culture through peer-led initiatives and visible endorsement by respected colleagues to enhance motivation and engagement.

^a^AI: artificial intelligence.

Furthermore, the influence of social context, particularly perceptions of colleagues’ attitudes, suggests that leveraging peer support and fostering a culture of openness toward innovation may play a role in facilitating adoption. Finally, embedding AI-related content into medical education and continuing professional development may support sustained engagement, particularly among groups less likely to adopt AI initially. These insights, while preliminary, could inform more inclusive and context-sensitive implementation strategies in future work.

### Future Research Directions

Future research could extend our findings through longitudinal studies that track real-world AI adoption over time, as well as intervention studies evaluating tailored support strategies. Comparative research across countries and medical specialties could further clarify how structural, educational, and cultural factors influence implementation [22,38]. In our sample, differences in access, training, and institutional support between users and nonusers highlight the need for flexible, context-sensitive implementation strategies. A governance framework, such as that proposed by Hassan et al [27], may support this by addressing trust-related barriers through structured oversight across the AI lifecycle and by informing policy and institutional priorities that enable more equitable access, investment, and training.

Our findings also support recent calls to expand AI education across the medical training pipeline. For example, Weidener and Fischer [39] emphasized the integration of AI ethics and practical skills in undergraduate curricula. Similarly, Wartman and Combs [40] argued that medical education must evolve beyond the information age to meaningfully prepare future clinicians for AI-enabled practice. Training physicians in AI as part of undergraduate and continuing medical education may enhance adoption by improving relevant skills, confidence, and preparedness for clinical integration, particularly among younger cohorts already engaging with AI informally [39].

Future research could also explore patient perspectives, particularly regarding data privacy, consent, and trust in AI-assisted diagnostics, to support ethical and patient-centered implementation. Although our study focused on physicians, these concerns are especially relevant in breast cancer screening, where patient acceptance may hinge on transparency, perceived fairness in algorithmic decision-making, and how AI is integrated into clinical workflows [41-43].

### Strengths and Limitations

A key strength of this study is its mixed methods design, which enabled triangulation of quantitative findings with qualitative insights. This approach allowed for a comprehensive understanding of AI adoption by comparing users and nonusers, and by identifying psychological, technical, and social drivers of adoption.

However, some limitations must be acknowledged. First, the sample was relatively small and limited to Austria, which may affect generalizability. Second, the cross-sectional design captures perceptions at a single point in time and does not allow for causal inference. Longitudinal research is needed to track evolving attitudes and behaviors. Third, reliance on self-reported data introduces potential bias, which could be addressed in future studies through objective usage metrics. Fourth, although items were adapted from validated TAM instruments and pilot tested for clarity, formal psychometric validation was not performed. The brief attitude measure and exploratory design, therefore, warrant cautious interpretation of these findings.

Regarding the quantitative analyses, the small sample size and the number of predictors in the regression models may limit statistical power and the stability of estimates. To address these concerns, we conducted extensive sensitivity and robustness checks, including alternative codings of barriers and facilitators, reduced models including only the main predictors, and ordered logistic regressions to account for the ordinal nature of the outcomes. These analyses indicate that the direction and magnitude of the observed effects are largely robust, although statistical significance varies slightly across specifications. Finally, recruitment via professional networks and social media groups meant that the total number of physicians reached was unknown, and a formal response rate could not be calculated. This may introduce self-selection bias and limit the generalizability of the findings beyond physicians with an interest or experience in AI. Despite these limitations, our study offers valuable exploratory insights into the factors influencing AI adoption in breast cancer diagnosis and provides a foundation for future implementation research and policy development.

### Conclusions

This study provides timely insight into AI adoption for breast cancer diagnostics among Austrian physicians and highlights several key contributions. First, it focuses on a specific clinical use case (breast cancer diagnostics) within a national health care context, extending prior work that has often examined AI adoption in radiology more broadly. Second, by comparing physicians with and without hands-on AI experience, it shows that barriers, facilitators, and support needs differ by level of AI exposure.

Third, the findings suggest that AI adoption may be better understood as a staged process rather than a binary decision. Early uptake appears to depend mainly on access and training, whereas later-stage use is more affected by workflow integration and trust in AI outputs. This refines existing TAMs by highlighting the role of implementation context across stages of engagement.

Finally, the results have practical implications for implementation. Investment in targeted training, organizational support, and peer-enabled learning may support more equitable adoption. Strategies that account for differences by experience level and demographic factors may be particularly important for achieving sustainable and responsible integration of AI in breast cancer diagnostics.

## Data Availability

Due to ethical restrictions related to participant confidentiality, the data supporting the findings of this study are not publicly available. However, anonymized data may be made available upon request to the corresponding author.

## Appendix

Multimedia Appendix 1 English translation of the questionnaire and mapping of questionnaire items to the analyses and reporting presented in the main manuscript.

Multimedia Appendix 2 Qualitative categories, codes, and illustrative quotes relating to barriers and facilitators of artificial intelligence adoption in breast cancer diagnostics.

Multimedia Appendix 3 Additional regression analyses and diagnostics, including descriptive statistics, model diagnostics, robustness checks, ordered logistic regression models, and reduced regression models predicting attitudes, intentions, and likelihood of future artificial intelligence use in breast cancer diagnostics.

## Abbreviations

AI: artificial intelligence
TAM: technology acceptance model
UTAUT: Unified Theory of Acceptance and Use of Technology
