# Supplementary material for: Barriers, Facilitators, and Intention to Use AI for Breast Cancer Diagnosis: Mixed Methods Study Among Austrian Physicians With and Without AI Experience
Source: J Med Internet Res. 2026 Jun 9;28:e80274. doi: 10.2196/80274 (PMC13291731; doi:10.2196/80274)
Supplement: Multimedia Appendix 1 [file jmir_v28i1e80274_app1.docx]

# Supplementary File 1

This supplementary file provides the final questionnaire (English translation) and a mapping of each questionnaire item to the analyses or reporting presented in the main manuscript.

**Questionnaire and Mapping to Analysed Variables**

The table below provides an overview of each questionnaire section and its corresponding analysis or reporting in the study. The English translation of the final questionnaire items is provided thereafter.

| **Questionnaire Section** | **Analysis/Reporting in Paper** | **Comment** |
| --- | --- | --- |
| **1. Consent** | No | Ethical requirement, mentioned under recruitment. |
| **2. Socio-demographics (age, gender, specialty, years of clinical experience etc.)** | Described in Table 1 (Descriptive Statistics) | Used as predictors in regression (e.g., Age 50+). |
| **3. Awareness and Current Use of AI** | Reported in Results (Awareness and Current Use), Open-ended responses informed qualitative themes. | Current use used for group comparisons (Users vs non-users). |
| **4. Knowledge, Skills, and Attitudes of Colleagues** | Regression Analysis: "Skills for Using AI", "Colleagues consider AI important", "Colleagues' opinion importance" | Key predictors in regression models (Table 5). |
| **5. Attitudes Toward AI** | Regression dependent variable: "Attitude toward AI" | Measured via composite mean score, 7-point Likert scale. |
| **6. Perceived Usefulness** | Regression Analysis | Used as a predictor in regression |
| **7. Perceived Barriers to AI Adoption** | Quantitative and Qualitative analysis (Tables 3) + Regression (Barrier Index) | For the regression, a dummy variable was created: "≥2 barriers" vs "<2 barriers"  Qualitative responses pertaining to barriers |
| **8. Perceived Facilitators to AI Adoption** | Quantitative and Qualitative analysis (Tables 2) + Regression (Facilitator Index) | For the regression a dummy variable created: "≥2 facilitators" vs "<2 facilitators  Qualitative responses pertaining to facilitators |
| **9. Intention to Use AI** | Regression dependent variable: "Intention to use AI" | Measured via single-item Likert. |
| **10 Motivation for Future AI Use** | Qualitative categories | Qualitative responses pertaining to barriers and facilitators |
| **11. Additional Comments** | Qualitative contextualization | Supplementary qualitative insights. |

# Questionnaire (English Translation)

## 1. Consent

I confirm that I have read the information sheet for this study, had the opportunity to ask questions, understand the purpose and extent of my participation, and voluntarily agree to participate.
Options: Yes I consent / No I do not consent

## 2. Sociodemographic Information

2.1 What is your age?

2.2 What is your gender? (Male / Female / Other)

2.3 What is your highest academic qualification?

2.4 What is your current position? (Private Practice / Junior Hospital Doctor / Senior Hospital Doctor / Department Head / Other)

2.5 In which municipality are you primarily working?

2.6 What is your medical specialty?

2.7.

**What is your area of work?**▢ Radiography/Mammography (1)
▢ Ultrasound (2)
▢ Angiography/Fluoroscopy (3)
▢ CT (4)
▢ MRI (5)
▢ PET/Nuclear (6)
▢ Hybrid Imaging (7)
▢ DXA (8)
▢ Experimental Imaging (9)
▢ Optical Imaging (10)
▢ Other. Please specify (11)

2.7 Which imaging modalities do you work with? (Mammography, Ultrasound, MRI, CT, etc.)

## 3. Awareness and Current Use of AI

3.1 Were you aware of the use of Artificial Intelligence for breast cancer diagnostics?
 (Yes / No)

o Yes, I am aware (1) No, I am not aware (2)

3.2 Are you currently using AI for breast cancer diagnostics? (Yes / No)

3.3 If yes, since when and how often do you use AI? Please briefly describe your previous use and/or experience with Artificial Intelligence for breast cancer diagnostics. (Open-ended)

3.4 If no, why have you not used AI for breast cancer diagnostics? (Open-ended)

## 4. Knowledge, Skills, and Social Influence

Do you personally have the necessary skills to use AI in breast cancer diagnostics? (Yes / No - Please specify skills missing)

How important is your colleagues' opinion about AI use to you? (0 = Not important at all to 6 = Very important)

Do your colleagues consider AI tools important for breast cancer diagnostics? (0 = Not at all to 6 = Very much)

## 5. Attitudes Toward AI

Please indicate your general attitude towards using AI in breast cancer diagnostics. (0 = Very Negative to 6 = Very Positive)

Please indicate your agreement with the following statement: (0 = Strongly Disagree to 6 = Strongly Agree)

• AI will improve the quality of breast cancer diagnostics.

## 6. Perceived Usefulness of AI

How useful do you consider the use of AI for breast cancer detection?
o 0 – Not useful at all (1)
o 1 (2)
o 2 (3)
o 3 (4)
o 4 (5)
o 5 (6)
o 6 – Very useful (7)

## 7. Perceived Barriers to AI Adoption

What barriers limit or prevent your use of AI for breast cancer diagnostics? (Tick all that are relevant)

☐ No barriers (1)

☐ AI is not available at my workplace for breast cancer diagnostics (2)

☐ High costs (3)

☐ Lack of user-friendliness or operability (4)

☐ Difficult technical integration into existing systems (5)

☐ Ensuring data protection (6)

☐ IT security (7)

☐ Lack of knowledge to use AI for breast cancer diagnostics (8)

☐ Lack of effectiveness (9)

☐ Please specify any other barriers (10): ________________________________

For participants who have NOT used AI: What are relevant barriers that may prevent future use of AI for breast cancer diagnostics? (Tick all that are relevant)

☐ No barriers (1)

☐ AI is not available at my workplace for breast cancer diagnostics (2)

☐ High costs (3)

☐ Lack of user-friendliness or operability (4)

☐ Difficult technical integration into existing systems (5)

☐ Ensuring data protection (6)

☐ IT security (7)

☐ Lack of knowledge to use AI for breast cancer diagnostics (8)

☐ Lack of effectiveness (9)

☐ Please specify any other barriers (10): ________________________________

## 8. Perceived Facilitators to AI Adoption

What advantages or benefits do you see in using AI for breast cancer diagnostics? (Multiple choice and open-ended text)

8a. For participants who HAVE used AI: What benefits has the use of AI in early breast cancer diagnostics brought you?

☐ No benefits (1)

☐ Faster task completion (2)

☐ More accurate diagnostics (3)

☐ I work more effectively with AI (4)

☐ Quality improvement of my work through better patient care (4)

☐ I am more productive in my work with AI (15)

☐ My work has become easier with AI (6)

☐ I have more control over my workflow with AI (7)

☐ I feel less stressed in my professional life with AI (8)

☐ Please describe any additional benefits (9): _________________

8b. For participants who have NOT used AI: What benefits could the use of AI in early breast cancer diagnostics bring you?

☐ No benefits (1)

☐ Faster task completion (2)

☐ More accurate diagnostics (3)

☐ I work more effectively with AI (4)

☐ Quality improvement of my work through better patient care (4)

☐ I am more productive in my work with AI (15)

☐ My work has become easier with AI (6)

☐ I have more control over my workflow with AI (7)

☐ I feel less stressed in my professional life with AI (8)

☐ Please describe any additional benefits (9): _________________

8c. Additional Question for All Participants:
What additional, not yet mentioned, specific benefits do you expect AI applications could bring to breast cancer diagnostics in the future?

## 9. Intention to Use AI and Likelihood of Future Use

How likely are you to use AI in breast cancer diagnostics in the future? (0 = Very Unlikely to 6 = Very Likely)

I intend to use AI in the future if possible. (0 = Strongly Disagree to 6 = Strongly Agree)

## 10. Motivation for Future AI Use

What factors would motivate you to adopt AI tools for breast cancer diagnostics? (Open-ended)

## 11. Additional Comments

Please share any additional thoughts or comments regarding AI in breast cancer diagnostics. (Open-ended)
