# Supplementary material for: Barriers, Facilitators, and Intention to Use AI for Breast Cancer Diagnosis: Mixed Methods Study Among Austrian Physicians With and Without AI Experience
Source: J Med Internet Res. 2026 Jun 9;28:e80274. doi: 10.2196/80274 (PMC13291731; doi:10.2196/80274)
Supplement: Multimedia Appendix 2 [file jmir_v28i1e80274_app2.docx]

## **Supplementary File 2**

| Table S1 and Table S2 provide illustrative quotes for qualitative codes derived from open-ended responses. **Table S1. Qualitative Categories and Illustrative Quotes Relating to Facilitators**  \| **Qualitative Category** \| **Qualitative Code** \| **Illustrative Quote** \| \| --- \| --- \| --- \| \| Improving Diagnostic Confidence and Trust in Accuracy \| More confidence in diagnostics \| I feel more secure in my diagnostic decisions. \| \| Improving Diagnostic Confidence and Trust in Accuracy \| Trust in AI as a second opinion \| It facilitates accuracy and validation through double verification of my diagnosis when I am not sure. \| \| Improving Diagnostic Confidence and Trust in Accuracy \| Improved diagnostic precision \| It improves patient care through better diagnostic precision. The quality of work has improved. \| \| Improved Efficiency as a Catalyst for Enhanced Quality of Care \| Faster diagnostics by automating routine tasks \| AI lets me focus on patients rather than routine diagnostics. \| \| Improved Efficiency as a Catalyst for Enhanced Quality of Care \| Automatic workflow integration \| The use of AI is integrated into the software and does not require conscious effort from my side. \| \| Improved Efficiency as a Catalyst for Enhanced Quality of Care \| Freeing up resources for complex cases and patient care \| Saves time on diagnostics, allowing me to focus on complex cases which improves quality of patient care. \| \| AI as a Catalyst for Continuous Learning and Professional Growth \| Using AI to educate resident physicians \| As a training tool, AI transforms routine diagnostics into teachable moments. \| \| AI as a Catalyst for Continuous Learning and Professional Growth \| AI as a clinical coaching tool \| AI tools could serve as a clinical coach for residents, providing tailored feedback and helping them improve diagnostic skills. \| \| Reducing Stress and Enhancing Well-being \| Reducing administrative burden \| Relief for support staff through handling image post-processing and preparing data for diagnosis. \| \| Reducing Stress and Enhancing Well-being \| AI reduces perceived workload \| Facilitates diagnosis and reduces workload. \| \| Reducing Stress and Enhancing Well-being \| Reduced pressure during busy shifts \| Use of AI decreases the pressure on me during busy shifts. \| |
| --- | --- | --- | --- | --- | --- | --- | --- | --- | --- | --- | --- | --- | --- | --- | --- | --- | --- | --- | --- | --- | --- | --- | --- | --- | --- | --- | --- | --- | --- | --- | --- | --- | --- | --- | --- | --- |

### **Table S2. Qualitative Categories and Illustrative Quotes Relating to Barriers**

| **Qualitative Category** | **Qualitative Code** | **Illustrative Quote** |
| --- | --- | --- |
| Access, Cost, and Integration Challenges | High costs making AI unaffordable | AI seems too costly to implement here. |
| Access, Cost, and Integration Challenges | Lack of availability at the workplace | The software is not available in the department. |
| Access, Cost, and Integration Challenges | Technical integration challenges | AI doesn’t align well with our current software. |
| Usability and Training Needs | Limited resources for training | It takes time to learn AI software, and training is limited. |
| Usability and Training Needs | Lack of knowledge and experience using AI | Easy handling and training opportunities would make adoption easier. |
| Data Privacy and IT Security Concerns | Patient data protection and privacy | AI brings up data protection issues for patients. |
| Data Privacy and IT Security Concerns | Security risks with AI use | Potential data security risks associated with using AI in clinical diagnostics. |
| Workflow Disruption and Limited Trust | Lack of workflow compatibility | It takes time to reconfigure clinical workflows and practices. |
| Workflow Disruption and Limited Trust | Lack of trust in AI as a sole decision maker | I rely on my own judgment to make the final decision. |
