# Supplementary material for: Barriers, Facilitators, and Intention to Use AI for Breast Cancer Diagnosis: Mixed Methods Study Among Austrian Physicians With and Without AI Experience
Source: J Med Internet Res. 2026 Jun 9;28:e80274. doi: 10.2196/80274 (PMC13291731; doi:10.2196/80274)
Supplement: Multimedia Appendix 3 [file jmir_v28i1e80274_app3.docx]

# **Supplementary File 3 - Additional Regression Analyses and Diagnostics**

This supplementary file provides additional analytical details supporting the regression analyses reported in the main manuscript. It includes (1) descriptive statistics for all variables used in the regression models (Table S1), (2) regression diagnostics assessing model assumptions, (3) robustness checks using alternative codings of facilitator and barrier variables, (4) ordered logistic regression models accounting for the ordinal nature of outcome variables, and (5) reduced models focusing on key predictors only. These analyses were conducted to ensure the robustness and transparency of the findings while maintaining a concise presentation in the main text.

## Descriptive Statistics of Regression Variables

| **Table S1. Descriptive Statistics of Variables Used in the Regression Model Predicting Attitudes, Intentions, and Likelihood of Future AI Use in Breast Cancer Diagnostics** | | | |
| --- | --- | --- | --- |
| Variable | Description or Scale | % or Mean | SD |
| Gender | Female | 21.95% | 0.42 |
| Age | Aged 50 and older | 63.41% | 0.49 |
| Current use of AI in breast cancer diagnostics | Currently using AI | 56.10% | 0.50 |
|  | Not using AI | 43.90% | 0.50 |
| Facilitators | Mentioned two or more  facilitators | 60.98% | 0.49 |
|  | Mentioned at most one facilitator | 39.02% | 0.49 |
| Barriers | Mentioned two or more barriers | 63.41% | 0.49 |
|  | Mentioned at most one barrier | 36.59% | 0.49 |
| Colleagues consider AI important¹ | Likert scale: 1 (Not at all) to 7 (Very important) | M = 4.78 | 1.31 |
| Importance of colleagues' opinion² | Likert scale: 1 (Not at all) to 7 (Very important) | M = 4.41 | 1.86 |
| General attitude toward AI³ | Likert scale: 1 (Very negative) to 7 (Very positive) | M = 5.49 | 1.21 |
| Intention to use AI in the future⁴ | Likert scale: 1 (Strongly disagree) to 7 (Strongly agree) | M = 5.29 | 1.75 |
| Likelihood of using AI in the future⁵ | Likert scale: 1 (Very unlikely) to 7 (Very likely) | M = 4.98 | 1.89 |

| **Note.**¹ Mean perception of how important colleagues consider AI; 23 participants (56.10%) indicated it was slightly important, important, or very important. ² Mean perceived importance of colleagues’ opinions about AI; 21 participants (51.20%) rated this as slightly important, important, or very important. ³ Mean general attitude toward AI in breast cancer diagnostics; 35 participants (85.37%) reported their attitude as slightly positive, positive, or very positive. ⁴ Mean intention to use AI in the future; 31 participants (75.61%) slightly agreed, agreed, or strongly agreed. ⁵ Mean perceived likelihood of future AI use in breast cancer diagnostics; 26 participants (63.41%) rated it as slightly likely, likely, or very likely. |
| --- |

## Regression Diagnostics

We conducted regression diagnostics for all three outcome variables (attitudes toward AI, intention to use AI, probability of using AI). Multicollinearity was not a concern: mean VIF = 1.41, max VIF = 1.67.

Regarding residual distributions, Shapiro–Wilk tests indicated no departures from normality (attitudes: W = 0.98, p = 0.831; intention to use: W = 0.98, p = 0.679; probability of using: W = 0.99, p = 0.957). Visual inspection of residual histograms and Q–Q plots further confirmed that residuals closely followed a normal distribution.

Tests for heteroskedasticity yielded mixed results. For attitudes toward AI, neither the Breusch–Pagan test (p = 0.533) nor White’s test (p = 0.547) indicated heteroskedasticity. For intention to use AI, the Breusch–Pagan test suggested potential heteroskedasticity (p = 0.037), whereas White’s test did not (p = 0.341). Similarly, for probability of using AI, the Breusch–Pagan test indicated heteroskedasticity (p = 0.002), but White’s test did not (p = 0.660). All regressions were estimated with heteroskedasticity-robust (Huber–White) standard errors, ensuring valid inference.

Overall, regression diagnostics confirm that the assumptions of linear regression were reasonably met.

Robustness Checks: Coding of Facilitator and Barrier Indicators

In the main regressions, facilitator and barrier indicators were coded as 1 if respondents reported two or more facilitators or barriers, and 0 otherwise. This choice avoids extremely unbalanced categories.

We conducted robustness checks using two alternative codings:

1. Any/None dummy – coded as 1 if respondents reported any facilitator/barrier, 0 otherwise.
2. Continuous sum-score – total number of facilitators or barriers reported.

Results:

- Facilitators: Positive effects of facilitators on attitudes, intention to use, and perceived probability of use remain consistent in direction across all specifications. Significance levels generally hold for the Any/None dummy. When using the continuous sum-score, facilitators remain significant for attitudes and intention to use, while the effect on probability of use becomes non-significant.
- Barriers:
  - Attitudes: Negative effects of barriers remain significant for both alternative codings.
  - Intention to use: In the Any/None dummy coding, the negative effect of barriers is slightly less significant (p = 0.053 vs. p = 0.047 in the main model), remaining meaningful at a liberal threshold (p < 0.1). Using the continuous sum-score, the effect is no longer statistically significant, though the estimated direction remains negative.
  - Probability of use: The negative effect of barriers remains robust with the Any/None dummy but is non-significant in the continuous sum-score specification.

Overall, these robustness checks indicate that the direction and general magnitude of the estimated effects of facilitators and barriers are stable, while statistical significance may vary slightly depending on coding choice. Detailed regression results for all alternative codings are provided in Tables S1-S2.

## Ordered Logistic Regression (ologit)

We estimated ordered logistic regression models for all three outcome variables (attitudes toward AI, intention to use AI, probability of using AI), using robust standard errors to account for the ordinal nature of the Likert-scale outcomes and the small sample size.

Findings:

- Facilitators: Positive effects of facilitators remain consistent across all outcomes. They are statistically significant for attitudes (p = 0.006), intention to use (p = 0.019), and probability of use (p = 0.001).
- Barriers: Negative effects of barriers are generally consistent with linear models.
  - For attitudes, the negative effect of barriers is slightly more significant in the ordered logistic model (p = 0.056 vs. p = 0.071 in the linear model), remaining meaningful at a liberal threshold of p < 0.1.
  - For intention to use, the negative effect is slightly less significant (p = 0.055 vs. p = 0.047 in the linear model), remaining meaningful at a liberal threshold of p < 0.1.
  - For probability of use, the negative effect remains highly significant (p = 0.001 vs. p = 0.000 in the linear model).
- Other covariates: Effects of skills, colleagues’ use, and age/female generally remain in line with linear models, with some variation in significance due to sample size.

Overall, these models confirm the robustness of our main findings: the direction and magnitude of facilitator and barrier effects are largely consistent with the linear regressions. Detailed results are presented in Table S3.

## Reduced Models (Main Predictors Only)

Given the relatively small sample size (n = 41) and the inclusion of 8–9 predictors in the main models, we estimated reduced models including only the main predictors: facilitators and barriers.

Findings:
The direction and magnitude of the coefficients for facilitators and barriers remain broadly consistent with the main models, indicating that observed effects are not driven by the inclusion of additional covariates. Significance levels vary slightly in some cases due to reduced model size, but the overall pattern of results remains stable. Detailed results are presented in Table S4.

**Table S2. Linear Regression Results Using Alternative Binary Coding of Facilitators and Barriers (≥1 vs. 0)**

| **Predictor** | **Attitude toward AI** | **Intention to use AI in future** | **Likelihood of using AI in future** |
| --- | --- | --- | --- |
| Facilitators (≥1) | 1.76 (0.57), p = .004* | 3.35 (0.67), p < .001* | 3.20 (0.61), p < .001* |
| Barriers (≥1) | -0.86 (0.28), p = .005* | -1.07 (0.53), p = .053 | -1.05 (0.56), p = .068 |
| AI user | -0.15 (0.35), p = .670 | 0.45 (0.43), p = .298 | 1.30 (0.51), p = .015* |
| Skills for using AI | 0.27 (0.32), p = .406 | 0.86 (0.37), p = .027* | 0.95 (0.42), p = .033* |
| Colleagues consider AI important | 0.43 (0.15), p = .006* | 0.50 (0.14), p = .001* | 0.09 (0.19), p = .617 |
| Colleagues’ opinion is important | 0.04 (0.08), p = .645 | -0.05 (0.08), p = .520 | 0.03 (0.11), p = .755 |
| Female | -0.19 (0.37), p = .614 | 0.05 (0.41), p = .901 | -0.83 (0.49), p = .101 |
| Age 50 and above | -0.84 (0.34), p = .018* | -0.36 (0.31), p = .257 | -0.43 (0.32), p = .196 |
| Constant | 1.68 (0.35), p < .001* | 0.53 (0.39), p = .186 | 0.21 (0.50), p = .683 |
| R² | 0.66 | 0.72 | 0.67 |

*Note.* Robust standard errors are shown in parentheses. * = indicates statistically significant predictors p ≤ .05. N=41

**Table S3. Linear Regression Results Using Continuous Sum Scores for Facilitators and Barriers**

| **Predictor** | **Attitude toward AI** | **Intention to use AI in future** | **Likelihood of using AI in future** |
| --- | --- | --- | --- |
| Facilitators (sum) | 0.28 (0.06), p < .001* | 0.22 (0.10), p = .043* | 0.17 (0.12), p = .173 |
| Barriers (sum) | -0.19 (0.07), p = .011* | -0.06 (0.12), p = .635 | -0.31 (0.13), p = .023* |
| AI user | 0.10 (0.24), p = .684 | 0.99 (0.55), p = .080 | 1.65 (0.59), p = .008* |
| Skills for using AI | 0.56 (0.24), p = .029* | 0.93 (0.52), p = .080 | 1.02 (0.51), p = .055 |
| Colleagues consider AI important | 0.27 (0.13), p = .048* | 0.40 (0.16), p = .019* | 0.04 (0.20), p = .828 |
| Colleagues’ opinion is important | 0.05 (0.06), p = .384 | -0.05 (0.08), p = .579 | 0.07 (0.11), p = .561 |
| Female | -0.05 (0.33), p = .887 | 0.14 (0.45), p = .757 | -0.61 (0.53), p = .262 |
| Age 50 and above | -0.97 (0.26), p = .001* | -0.60 (0.39), p = .139 | -0.62 (0.35), p = .088 |
| Constant | 1.20 (0.33), p = .001* | -0.15 (0.62), p = .805 | 0.22 (0.71), p = .763 |
| R² | 0.73 | 0.59 | 0.60 |

*Note.* Robust standard errors are shown in parentheses. * = indicates statistically significant predictors p ≤ .05. N=41

**Table S4. Ordered Logistic Regression (ologit) Results for Main Predictors (≥2 Facilitators/Barriers), Robust Standard Errors**

| **Predictor** | **Attitude toward AI** | **Intention to use AI in future** | **Likelihood of using AI in future** |
| --- | --- | --- | --- |
| Facilitators (≥2) | 2.24 (0.81), p = .006* | 2.16 (0.92), p = .019* | 3.14 (0.93), p = .001* |
| Barriers (≥2) | -1.52 (0.80), p = .056 | -1.45 (0.75), p = .055 | -3.19 (0.93), p = .001* |
| AI user | -0.34 (1.01), p = .739 | 0.98 (0.98), p = .315 | 2.32 (0.97), p = .016* |
| Skills for using AI | 0.90 (0.79), p = .251 | 1.74 (0.76), p = .022* | 2.36 (0.91), p = .009* |
| Colleagues consider AI important | 0.97 (0.47), p = .040* | 0.90 (0.32), p = .005* | 0.11 (0.33), p = .734 |
| Colleagues’ opinion is important | 0.22 (0.24), p = .361 | -0.02 (0.18), p = .895 | 0.11 (0.18), p = .545 |
| Female | -0.80 (1.14), p = .483 | 0.09 (0.91), p = .922 | -1.84 (0.89), p = .039* |
| Age 50 and above | -2.90 (0.91), p = .001* | -1.38 (0.85), p = .103 | -1.64 (0.74), p = .027* |

*Note.* Robust standard errors are shown in parentheses. * = indicates statistically significant predictors p ≤ .05. N=41

**Table S5. Reduced Linear Regression Models (Main Predictors Only, Robust Standard Errors)**

| **Predictor** | **Attitude toward AI** | **Intention to use AI in future** | **Likelihood of using AI in future** |
| --- | --- | --- | --- |
| Facilitators (≥2) | 0.84 (0.39), p = .038* | 1.44 (0.64), p = .032* | 1.46 (0.53), p = .009* |
| Barriers (≥2) | -0.77 (0.40), p = .061 | -1.38 (0.59), p = .025* | -2.10 (0.50), p = .000* |
| Constant | 1.46 (0.33), p < .001* | 1.29 (0.44), p = .005* | 1.42 (0.40), p = .001* |

*Note.* Robust standard errors are shown in parentheses. * = indicates statistically significant predictors p ≤ .05. N=41
